# Regarding the Yin and Yang of Precision Cancer- Screening and Treatment: Are We Creating a Neglected Majority?

**DOI:** 10.3390/ijerph16214168

**Published:** 2019-10-29

**Authors:** Colleen M. McBride, Yue Guan, Jennifer L. Hay

**Affiliations:** 1 Behavioral Science and Health Education Department, Rollins School of Public Health Emory University, Atlanta, GA 30322, USA; yue.guan@emory.edu; 2 Department of Psychiatry and Behavioral Sciences, Memorial Sloan Kettering Cancer Center, New York City, NY 10022, USA; hayj@MSKCC.ORG

**Keywords:** precision cancer screening and treatment, negative results, health communication, population-based family history screening, germline testing for hereditary cancer syndromes, tumor testing for targeted cancer therapies

## Abstract

In this commentary, we submit that the current emphasis of precision cancer screening and treatment (PCST) has been to provide and interpret the implications of “positive” screening results for those deemed to be at greatest risk for cancer or most likely to benefit from targeted treatments. This is an important, but proportionately small target group, regardless of the cancer context. Overlooked by this focus is the larger majority of those screened who receive “negative” results. We contend that for optimal dissemination of PCST, the complement of positive and negative results be viewed as an inseparable yin–yang duality with the needs of those who receive negative screening results viewed as important as those deemed to be at highest risk or derive targeted treatment benefit. We describe three areas where communication of negative PCST results warrant particular attention and research consideration: population-based family history screening, germline testing for hereditary cancer syndromes, and tumor testing for targeted cancer treatment decision-making. Without thoughtful consideration of the potential for negative results to have psychological and behavioral influences, there is a potential to create a “neglected majority”. This majority may be inclined to misinterpret results, disseminate inaccurate information to family, dismiss the credibility of results, or become disillusioned with existing medical treatments.

## 1. Introduction

The availability of a growing compendium of genomics-informed screening and treatment options now illustrate both the opportunities and challenges of implementing precision public health and medicine. On the public health front, several national organizations concur that there is strong evidence to support population screening to identify individuals and families at high risk for inherited cancer syndromes [1]. Achieving the public health benefit of such screening would involve implementation of widespread family history assessment in primary care and through national and community programs. The availability of scientifically validated, brief screening tools now make it feasible to triage high-risk individuals to appropriate genetic counseling (e.g., [1]). Those tested and found to carry germline mutations associated with cancer susceptibility genes can be referred to preventive and life-saving treatment options. Concurrently, advances in molecular and genetic tumor profiling can increasingly identify actionable genetic mutations to guide targeted and individualized cancer therapies to reduce the growth and spread of cancer [2].

In each of these screening contexts, those identified at greatest cancer risk (e.g., suggestive family history, confirmed mutation carrier) or with tumor characteristics most likely to respond to targeted therapy stand to benefit. Accordingly, those at highest risk or most likely to benefit from targeted treatment have been the focus when characterizing the potential of precision public health and medicine advances (e.g., [3,4]). Yet, the potential impact of this screening for the vast majority of those found not to be at high risk or unlikely to benefit from target therapies is largely not considered.

We believe that the ancient Chinese philosophy of yin–yang has an important lesson for broad implementation of genomics-informed “precision” cancer screening and treatment (PCST). The philosophy highlights the abiding truth that opposite forces are actually interconnected, and dependent on each other for definition and clarity. In genomic screening scenarios, this duality comprises “high risk,” indicating there is genetic influence on susceptibility to a health outcome or a treatment; whereas “not at high risk”, or negative results indicate that the gene(s) of interest is not a critical contributing factor. Based on the yin–yang principle, these two results include and define each other; just as “high” defines and includes “low”, positive and negative genomic screening results are a similar inseparable duality. All genomic screening—whether family history based, germline genetic testing, or tumor testing—embody this inherent duality. As such, we contend that for optimal dissemination of PCST, high-risk and negative results be viewed as an inseparable duality such that the needs of those who receive negative screening results are as important as those deemed to be at highest risk or benefit.

Currently, the majority of translation research in PCST focuses solely on the yang of “high-risk” results. As evidence, innumerous observational and experimental studies have evaluated strategies to effectively interpret information for those found to be at high risk for carrying genetic mutations that are associated with negative health outcomes. Similarly, efforts to motivate uptake of preventive behaviors almost exclusively have focused on the benefits and risks of PCST for identifying the yang side of increased susceptibility. Often these studies rightly argue for the significance of high-risk results to compel action, such as seeking prevention options or treatment adherence.

In contrast, there is little research focused on effective communication and appropriate follow-up for those deemed not to be at increased risk for mutations or unlikely to benefit from tailored treatment [5]. The irony is that most individuals (upwards of 50%) who participate in PCST will receive these negative or uninformative findings. Thus, the great preponderance of those screened will have to integrate negative findings into a view of their overall health, and in some cases, that of their family members; these individuals receive little or no guidance on the implications of their findings for cancer risk generally.

In this commentary, we suggest that optimal dissemination of PCST will require development of best practices for providing and interpreting the implications of receiving negative results. We use the term “best practices” to mean suitable standard procedures that have the potential to be widely adopted [6]. We describe three areas where communication of negative PCST results warrant particular attention and research consideration: population-based family history screening, germline testing for hereditary cancer syndromes, and tumor testing for targeted cancer treatment decision-making (see Table 1). We begin with discussion of the underpinnings of the “yang” emphasis to make the case that in the absence of considering the yin–yang duality, we risk creating a neglected majority. We use “neglected majority” as a shorthand to encompass emerging empirical and conceptual support that in deploying PCST: (1) the majority tested will receive negative results; (2) there is potential that these results could lead to iatrogenic psychological and behavioral impacts that include misinterpretation and dismissal of results, dissemination of inaccurate information to family, and disillusionment with existing medical treatments. We also may miss an opportunity to leverage these negative results to foster positive psychological and behavioral outcomes and promote health for this majority.

## 2. Materials and Methods

### 2.1. How Did We Get to be So Yang Focused

The field of genetic screening and testing has roots in rare disease and prenatal contexts. In these contexts, the yang focus on high-risk screening results derives from the medical model where the goal is largely diagnostic; the goal is to identify an underlying cause (e.g., responsible gene influence) that informs treatment. High-risk PCST results have been well delineated, have established predictive validity and reliability, and point to options for medical solutions such as pregnancy termination, enhanced surveillance, and specific treatments. Accordingly, the U.S. Preventive Services Task Force and others view awareness of high-risk results to have significant lifesaving and prevention benefits [1].

By contrast, in the emerging landscape of broadening genomic screening for cancer susceptibility, as only 5–20% of those screened will be at high-risk; the “yin” group comprises the majority tested. To date, actions relating to the yin of negative results where hereditary susceptibility to cancer is unlikely often are handled perfunctorily, or not at all. Among many implicit assumptions is that negative screen results have no potential harm or benefit for health outcomes. Yin-result responses such as confusion, guilt, disappointment, or defensive processing and their impact on screening and treatment adherence are rarely considered.

### 2.2. Why Should We Care about The Yin-Side?

Communication and behavioral theories tell us that those receiving negative or uninformative results are likely to experience many cognitive and emotional responses that have not been adequately explored for their downstream influences on health outcomes [7,8]. Butterfield and colleagues (2019) also suggest that early adopters of genomic screening tend to perceive themselves to be at increased risk and are confident they can understand results. Yet, fewer than 20% were able to understand the nuance of a negative screening result [9]. The majority of these early adopters will receive a negative PCST result. Moreover, movement to genomic screening based on large gene panels for germline and tumor testing will increasingly challenge our notions of what even constitutes negative risk. This increasing heterogeneity of “negative” results will call for deeper consideration of what these results mean.

In the next sections, we highlight three genomic screening contexts to illustrate the importance of directing more research attention to inform best practices for managing the return of negative and inconclusive results.

### 2.3. Population-Based Family History Screening

The majority of those who undergo family history screening will receive negative results. For example, 85–90% of those screened for hereditary breast and ovarian cancer (HBOC) will screen negative, that is, be unlikely to carry a *BRCA1/2* mutation [10,11]. We can all agree it is vitally important that individuals who are not likely to carry a specific mutation understand that they remain at risk for breast cancer. In a recent pilot study, we found that well-educated women were not able to distinguish between their risk for a *BRCA1/2* mutation and their general breast cancer risk. Just one-third of women who were told that they were at low risk of carrying a *BRCA1/2* mutation understood that they remained at average risk for developing breast cancer [12]. Butterfield and colleagues (2019) reported similar levels of misunderstanding of nuanced information related to negative findings from a gene panel among a well-educated sample [9].

Widespread use of family history screening for hereditary cancers could have other iatrogenic psychological and behavioral effects if women misperceive negative results. Unfortunately, information-processing theories tell us this is quite likely. Emotions evoked by risk information can elicit self-protective motives to distance oneself, dismiss, or discount the information, particularly when results are unexpected [7,8,13]. These processes influence the extent to which the individual is likely to accept or reject the risk message as relevant, and likely to influence message acceptance, another component of understanding.

In the case of HBOC screening results, for example, women who underestimate their breast cancer risk may mistakenly interpret negative HBOC screen results to support this viewpoint, and may, therefore, be disinclined to pursue routine mammography. Alternatively, women who overestimate their risk may reject the validity of the screening results and seek mammograms at inappropriate intervals, thereby increasing their risk for false-positive results [14]. Moreover, our pilot work suggests that these misunderstandings may foster inaccurate communications to the broader family network [15]. Our pilot work also suggests that women who were annual mammography screeners and indicated any cancer worry were significantly less likely to accept negative mutation risk information based on a validated family history assessment [12].

Furthermore, population subgroups may have patterns of social experience that influence their responses to negative genetic screening results. Minority groups with experiences of discrimination in referrals and inadequate access to health services may be unwilling to accept the veracity of negative findings and the implications that they could reduce the frequency of mammography screening [16]. Indeed, we found that African American women who were routine mammography screeners were less likely to accept results indicating they were at low risk for carrying a *BRCA1/2* mutation than their white counterparts [12]. These responses may reduce their willingness to adhere to guideline recommendations for the appropriate frequency of mammogram screening. Unwillingness to adopt screening suggested by PCST risk results may undermine the potential for health care cost reductions suggested by precision public health.

### 2.4. Germline Testing for Hereditary Cancer Syndromes

Genetic counseling and testing to identify individuals who carry germline mutations in cancer susceptibility genes (e.g., *BRCA1* and *BRCA2*, DNA mismatch repair [*MMR*] genes, *APC*, *TP53*, etc.) are widely endorsed to guide decisions about cancer prevention and early detection [17]. However, hereditary cancer syndromes account for just 10% of all malignancies [17]. Notification of being mutation-negative in a family with a known mutation (aka “true negative”) can be very informative with clear follow-up recommendations.

The psychosocial and behavioral consequences of receiving negative germline genetic test results also may not be benign. Prior research suggests that many patients who received a negative test result for one syndrome (e.g., *BRCA* only) erroneously concluded that they did not carry a pathogenic mutation in a cancer susceptibility gene [18,19]. Negative genetic test results also may fail to reassure. For example, a study conducted 20 years ago found that one-third of women under 40 who tested negative for *BRCA1/2* mutations sought mammography screening within a year, despite it not being clinically recommended [20]. Similarly, 42% of those who tested negative for genes involved in familial adenomatous polyposis intended to undergo unnecessary bowel screening [21,22]. However, much of the research related to the implications of these misunderstandings was in the context of single gene testing and is quite dated.

Of additional importance is that a sizeable group of those tested will receive results that are “negative but uninformative” or “variant of uncertain significance” (VUS). These results elude straightforward interpretation and raise communication challenges for establishing best practices.

Those receiving VUS results may inaccurately perceive risk, experience uncertainty, distress and frustration, and make radical treatment decisions (e.g., prophylactic surgeries) [23,24,25,26,27]. Professional associations also endorse the need for timely updates to individuals and families with VUS as evolving genomic science provides greater clarification. Yet the growing volume of PCST test results may overburden the limited genetic services infrastructure, and there is no professional consensus on how and when to conduct updates [28].

Due to the limited representation of minority populations in cancer basic science and genetic testing uptake, minority groups are more likely to receive negative genetic test results such as VUS [29,30]. This may leave minority patients and their family members with heightened risk awareness and no clear course of action [31], and further entrench health disparities in cancer care.

Genetic testing in the context of research also raises concerns about the neglected majority. There is growing consensus that genetic test results with clinical utility collected in research studies be returned to study participants. To date, cancer genetic studies have predominantly focused on returning positive results that is, a mutation associated with increased cancer risks is identified [32]. However, negative results, with clinical utility and of interest to participants [33,34], are generally not disclosed or CLIA-verified.

Professional guidelines are moving toward offering multigene panel testing using expanded testing criteria. For example, the American Society of Breast Surgeons recommend all breast cancer patients receive panel testing [35]. This approach will undoubtedly increase the occurrence of negative results. There is a need to investigate new communication challenges around uncertainty, expectations and the amount and complexity of information.

### 2.5. Targeted Cancer Therapies

The use of a person’s genes and proteins to treat cancer is a cornerstone of precision medicine. Advances in the use of next-generation sequencing tests that identify actionable genetic mutations now inform “targeted cancer therapies”—agents that interfere with specific molecules (“molecular targets”) implicated in the growth, progression, and spread of cancer [2]. Such therapies are distinct from standard chemotherapies that act on all rapidly dividing cells, both cancerous and noncancerous. For example, targeted therapies to treat advanced melanoma by counteracting the effects of mutations in a *BRAF* oncogene gene are approved by the Food and Drug Administration [36]. Combination therapy with dabrafenib and trametinib (combining a *BRAF* inhibitor and *MEK* inhibitor) is now standard care for these patients [37,38]. Yet point mutations in the *BRAF* gene occur in only about 40% to 50% of melanomas [39], and make targeted treatments unavailable to half or more of advanced melanoma patients. Other approved targeted therapies include the human epidermal growth factor receptor 2 protein (HER2) and traztuzumab (Herceptin) expressed at high levels on the surface of some cancer cells can treat some breast and stomach cancers [40], and therapies for some forms of non-small cell lung cancer and colorectal cancer, among others [2]. As with melanoma, 50% or more of patients tested have no mutations found or alternations in actionable genes [2]. As such, despite the excitement and promise surrounding targeted therapies, only a subset of cancer patients can currently reap the benefits of these important advances.

Again, there is the potential for psychological and behavioral effects of receiving these negative results. For example, patients with advanced cancer who receive “negative” or “not actionable” tumor mutation findings may experience a number of emotional reactions that could influence treatment-related behaviors. Communication and behavioral theories would suggest these patients may experience disappointment, fatalism about cancer progression, or lack of confidence that standard, “non-targeted” treatments will be effective, all with potential to undermine adherence to cancer treatment (e.g., [41]). While a limited literature has begun to identify unmet patient expectations and needs regarding tumor testing (e.g., [42,43]), examination of these issues specifically among the majority group who are not eligible for novel tumor-based treatments has not been advanced. Rigorous research to consider the ideal content, framing, and timing of physician and staff communication to anticipate and address these reactions is needed to inform best practices.

## 3. Conclusions

Growing evidence supports the value of population-wide family history screening, mutation testing, and targeted cancer therapies, a.k.a., precision public health and medicine. We contend that the health potential of these efforts will not be achieved without thoughtful consideration of the yin–yang duality of genomic-informed screening. This will call upon us to anticipate the communication needs of the majority group who will receive an ever-increasing variety of negative results. Proactive consideration of communication challenges via a conceptually grounded research program could reduce the likelihood for negative psychological and behavioral effects, and maximize the potential for adherence to prevention, screening, and treatment recommendations. We have highlighted just a few of the scenarios that create opportunities now to begin this line of research so that genomic discoveries benefit all and do not undermine health nor deepen disparities.

## Figures and Tables

**Table 1:** Implications of negative results by level of precision screening.

Precision Screening	Results/Meaning	Implications	Recommendations
**Population family history-based screening**	**Negative** [i.e., individual’s family history is not suggestive of hereditary cancers]	• Low risk for hereditary cancers • Average or increased cancer risk based on other personal factors	• Standard prevention based on other personal factors
**Germline genetic testing**	**True negative** [i.e., individual has no cancer predisposing gene mutation in a family where another member(s) has tested positive]	• Low risk for hereditary cancers • Average or increased cancer risks based on other personal factors	• Standard prevention based on other personal factors
	**Uninformative** [i.e., individual has no cancer predisposing gene mutation in a family where another member(s) has tested negative or not been tested]	• Unknown risk for hereditary cancers • Individuals may have an inherited genetic abnormality	• More extensive genetic testing • Standard prevention based on other personal factors
	**Variant of uncertain significance** [i.e., individual has a gene change but its relationship with cancer is unclear]	• Unknown risk for hereditary cancers	• Periodic follow up with possible variant re-classification • Standard prevention based on other personal factors
**Tumor testing**	**Negative or uninformative** [i.e., a gene mutation associated with targeted cancer therapy benefit is not found in the tumor]	• Specific precision-based treatment not indicated	• Standard treatment

Examples of personal factors include age, gender, smoking, alcohol consumption, and personal medical history (e.g., chest radiation for Hodgkin Lymphoma, inflammatory bowel disease).
